# Navigating Alzheimer’s Disease Mouse Models: Age-Related Pathology and Cognitive Deficits

**DOI:** 10.3390/biom14111405

**Published:** 2024-11-05

**Authors:** Laura Maria De Plano, Alessandra Saitta, Salvatore Oddo, Antonella Caccamo

**Affiliations:** Department of Chemical, Biological, Pharmaceutical and Environmental Sciences, University of Messina, Viale F. Stagno d’Alcontres 31, 98166 Messina, Italy; lauramaria.deplano@unime.it (L.M.D.P.); alessandra.saitta@unime.it (A.S.); salvatore.oddo@unime.it (S.O.)

**Keywords:** transgenic mice, APP, tau, PS1, Aβ

## Abstract

Since the mid-1990s, scientists have been generating mouse models of Alzheimer’s disease to elucidate key mechanisms underlying the onset and progression of the disease and aid in developing potential therapeutic approaches. The first successful mouse model of Alzheimer’s disease was reported in 1995 with the generation of the PDAPP mice, which were obtained by the overexpression of gene coding for the amyloid precursor protein (APP). Since then, scientists have used different approaches to develop other APP overexpression mice, mice overexpressing tau, or a combination of them. More recently, Saito and colleagues generated a mouse model by knocking in mutations associated with familial Alzheimer’s disease into the APP gene. In this review, we will describe the most used animal models and provide a practical guide for the disease’s age of onset and progression. We believe that this guide will be valuable for the planning and experimental design of studies utilizing these mouse models.

## 1. Introduction

Currently, there are no cures or effective treatments for Alzheimer’s disease (AD), the most common neurodegenerative disorder. It is estimated that 7.2 million people in Europe and another 6.9 million in the US have AD [1,2]. This number is only destined to grow, given the increased life expectancy in industrialized countries and the lack of cures and effective treatments [1,2].

Individuals with AD are characterized by a distinct clinical trajectory that affects cognitive, behavioural, and functional domains. The hallmark clinical manifestation begins with subtle episodic memory impairments, particularly affecting recent memories, which gradually worsen over time. As the disease advances, deficits extend to other cognitive domains such as language (aphasia), visuospatial abilities (apraxia and agnosia), and executive functions. Overall, these changes lead to impaired judgment, problem-solving, and planning. In addition, AD patients show other behavioural disturbances such as depression, apathy, agitation, and irritability, which are more common in more advanced stages of the disease. In the later stages, patients experience profound disorientation, loss of independence in activities of daily living, and significant motor impairments [3,4,5].

Neuropathologically, the AD brain is characterized by the accumulation of neurofibrillary tangles, amyloid-β (Aβ) plaques, and an exaggerated neuroinflammatory response. Tangles consist of hyperphosphorylated tau, a microtubule-binding protein genetically associated with frontotemporal dementia with parkinsonism linked to chromosome 17 (FTDP-17). Tau is encoded by a gene on chromosome 17, which, through alternative splicing, produces six different isoforms. Tau is predominantly expressed in neurons, where it plays a critical role in stabilizing microtubules. Thus, it is crucial for axonal integrity and function, supporting the trafficking of organelles, vesicles, and proteins necessary for neuronal signalling. Under pathological conditions, tau can undergo hyperphosphorylation, leading to its dissociation from microtubules and the formation of insoluble tangles [6].

Plaques are primarily composed of a small peptide called Aβ, which is derived from the proteolytic cleavage of amyloid precursor protein (APP) by beta-site APP-cleaving enzyme 1 (BACE-1) and the gamma-secretase complex, where presenilin-1 (PS1) or presenilin-2 (PS2) serve as the catalytic subunits [7]. APP is a transmembrane protein abundantly expressed in neurons and is implicated in a variety of physiological processes, including synapse formation, neural plasticity, and cell signalling. APP can undergo a non-amyloidogenic processing which precludes the formation of Aβ [7].

While neuroinflammation is a consistent feature of the AD brain, it has only recently become recognized as a primary component of the disease process, following several genome-wide association studies (GWAS) that identified links between many inflammatory genes and AD [8,9,10]. It plays a dual role in AD progression. In the early stages, the brain’s immune cells, particularly microglia and astrocytes, respond to plaques and tangles by attempting to clear these aggregates through phagocytosis and the release of anti-inflammatory cytokines. However, chronic exposure to Aβ and tau leads to sustained activation of these glial cells, resulting in a prolonged inflammatory response. This chronic neuroinflammation is characterized by the excessive release of pro-inflammatory cytokines, chemokines, and reactive oxygen species, which exacerbate synaptic damage, neuronal death, and further promote plaque and tangle formation [11]. Numerous mechanisms at the centre of this feedback loop have been described in the literature [12,13].

The etiology of AD is attributed to both genetic and environmental factors [14]. In a few cases (<5%), known as early-onset familial AD (FAD), the disease is triggered by mutations in three key genes: APP, PSEN1, and PSEN2, all of which are involved in Aβ production [7]. In contrast, late-onset sporadic AD, which represents more than 95% of cases, is largely influenced by a combination of genetic risk factors and environmental influences. The apolipoprotein E (APOE) gene, particularly the APOE ε4 allele, is the strongest genetic risk factor for sporadic AD, influencing Aβ metabolism and clearance [15]. Beyond genetic predisposition, age remains the greatest risk factor for AD, with the prevalence of the disease increasing dramatically in individuals over 65. Other contributing factors include vascular health, diabetes, hypertension, and lifestyle aspects such as diet and physical activity [16,17]. Growing evidence also points to neuroinflammation, mitochondrial dysfunction, and impaired protein clearance pathways as critical components of the disease process [18,19,20]. The complex interplay of these genetic and environmental factors highlights the heterogeneity of AD, emphasizing the need for diverse model systems to study the disease’s mechanisms. Among these, transgenic mice are widely used in the field. Notably, all successful mouse models of Alzheimer’s disease express either mutant human Aβ or a humanized version of the APP gene. This is essential because mouse Aβ does not aggregate as readily as human Aβ due to structural differences in their peptide sequences. Specifically, mouse Aβ contains arginine, tyrosine, and histidine at positions 5, 10, and 13, respectively, whereas human Aβ has glycine, phenylalanine, and arginine at these positions. These substitutions likely reduce the self-aggregation propensity of mouse Aβ by altering interactions with metal ions necessary for aggregation [21,22].

## 2. Mouse Models of Alzheimer’s Disease

In this review, we describe the most commonly used mouse models of AD, selected based on citation frequency of the original publication detailing the model’s generation and its widespread distribution within the scientific community (Table 1). Specifically, we focus on establishing a timeline for the progression of the AD-like phenotype in these mice.

### 2.1. PDAPP Mice

**Figure 1:**
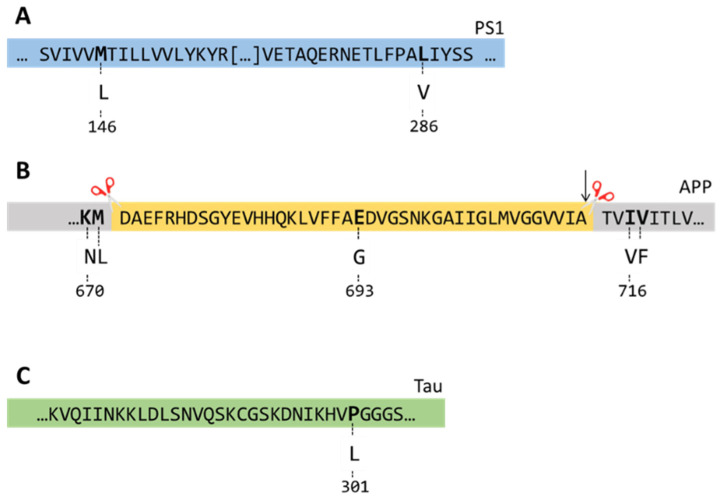
Schematic representation of the three genes used to generate the PDAPP, Tg2576, APP23, J20, CRND8, 3×Tg-AD, APP/PS1, 5×FAD, and APP^NL-G-F/NL-G-F^ mice. The mutations included in the PS1 (**A**), APP (**B**), and tau (**C**) genes are reported. The sequence in yellow in panel B represents Aβ.

The first successful mouse model of AD was developed by overexpressing human APP harbouring the Indiana mutation (V717F; Figure 1B) under the control of the PDGF promoter [23]. These mice were generated on a mixed genetic background of C57BL/6, DBA, and Swiss–Webster strains [23]. However, since their initial generation, several congenic lines have been created, showing notable effects on cognitive and neuropathological deficits.

While the APP transgene is well expressed in the cortex, hippocampus, hypothalamus, and cerebellum, the increase in Aβ levels is mainly observed in the hippocampus and cortex. Specifically, compared to 4-month-old mice, there was a 400- and 500-fold increase in Aβ levels in the hippocampus and cortex, respectively, in 18-month-old mice [24]. Aβ deposits in homozygous mice were also detected starting at 4 months of age in the cortex and hippocampus. By the time the mice reached 18 months of age, widespread Aβ deposits were present throughout both brain regions [24]. In contrast, in hemizygous mice, Aβ deposition was first apparent at 8 months of age in the cingulate cortex and at 12 months of age in the hippocampus. As with homozygous mice, hemizygous mice showed a significant increase in Aβ burden by the time they reached 18 months of age [25]. Extracellular Aβ accumulation is followed by neuroinflammation, which is first apparent at 6 months of age [23].

While these mice do not develop frank neuronal loss, a 31% reduction in the size of the cell bodies of TH-positive neurons in the dorsal central portion of the locus coeruleus has been reported [25,26]. In addition, a decrease in hippocampal spine density is first detected at 2 months of age, preceding Aβ deposition [27], but was not observed at 11 months of age [27]. Consistent with the decrease in spine density, deficits in long-term potentiation (LTP) were observed in 4–5-month-old mice, before the onset of frank Aβ plaques [28].

Homozygous and hemizygous PDAPP mice on the original background show reference and working memory impairments, as detected by the radial arm water maze, as early as 3 months of age [29,30]. These results were later confirmed using the Morris water maze (MWM) [31]. Since their development, the genetic background of the PDAPP mice has been modified, which has altered the timeline of cognitive deficits. Specifically, hemizygous PDAPP mice on a C57BL/6 background first show memory deficits at 14–16 months of age [32].

A major strength of the PDAPP mice is the gradual accumulation of Aβ throughout their lifespan. However, a significant drawback is the absence of overt neurodegeneration and neurofibrillary tangles.

### 2.2. Tg2576 Mice

The development and characterization of Tg2576 mice was first described in 1996. Since then, these mice have been widely used in the scientific community. Tg2576 mice overexpress human APP695 harbouring the Swedish mutation (K670N-M671L; Figure 1B) under the control of the hamster PrP promoter [33].

Brain Aβ levels, detected by sandwich ELISA, are first observed around 2–3 months of age and increase as the mice age [33,34]. Plaques are first detected at 7–8 months of age in the temporal cortex, and by 10–12 months of age, they are readily observed in the cortex and hippocampus. In mice older than 15 months, plaques are widespread in the cortex and hippocampus [33,34,35]. Microglia activation is detected starting at 10 months of age and is present around mature Aβ plaques [36].

While neuronal density and neuron number in CA1 are unaltered in 16-month-old mice [35], striking degeneration of the locus coeruleus has been reported to occur between 6.5 and 8 months of age [37]. Additionally, Golgi staining revealed a decrease in dendritic spine density in 4- and 11-month-old mice [27]. Interestingly, spine density was unaltered in 20-month-old mice [27].

The extent of synaptic deficits in Tg2576 mice has been reviewed elsewhere [38] and the results are not always consistent among publications. For example, Chapman and colleagues reported that hippocampal LTP was not altered in 2–8-month-old Tg2576 mice but it was severely impaired in 15–17-month-old mice [39]. In contrast, Fitzjohn and colleagues reported that while synaptic transmission was impaired in aging Tg2576 mice, LTP was preserved [40]. More recently, it has been reported that Tg2576 mice develope LTP deficits in the Schaffer collaterals; LTP was similar between transgenic and wild type mice when measured in the hippocampal mossy fibres [41].

Cognitive deficits appear across different domains, with spatial reference memory and working memory deficits beginning around 6 months of age and worsening as the mice age [42,43]. Indeed, robust impairments in the MWM and radial arm water maze (RAWM) have been reported in 8.5-month-old mice [42,43].

A major strength of Tg2576 mice is the consistency of their Aβ phenotype, which, as in PDAPP mice, develops progressively with age. However, a notable drawback is the lack of significant neurodegeneration and neurofibrillary tangles. From a practical standpoint, Tg2576 mice are also highly aggressive, which can make them challenging to handle.

### 2.3. APP23 Mice

In APP23 mice, the Thy1.2 promoter drives the expression of APP751 carrying the Swedish mutation (Figure 1B), leading to a 7-fold increase in APP expression over the endogenous gene [44]. While these mice were originally generated on a C57BL/6-DBA2 background, they have been backcrossed to C57BL/6 [45].

Soluble Aβ levels were significantly higher in the brains of 2-month-old APP23 mice compared to wild-type mice, and the difference between the two genotypes becomes more pronounced as the mice age [46]. In contrast, Aβ plaques are first detected in the cortex at 6 months of age and are present in high numbers and density throughout the brain in 2-year-old mice [44,47]. As in PDAPP and Tg2576 mice, neuroinflammation follows the development of Aβ plaques and is first apparent at 6 months of age [48].

An increase in phosphorylated tau, detected by western blot using the AT8 antibody, has been observed in 6- and 15-month-old mice [44]. Dystrophic neurites surrounding plaques were also associated with increased AT8 immunoreactivity [44,49].

CA1 neuronal loss, which correlated with plaque load, has been reported in 14- and 18-month-old mice [50]. Interestingly, 8-month-old APP23 mice have a higher number of neurons in the cortex compared to age-matched wild-type mice [45]. However, by 18 months, no difference was detected between the two groups [45,50].

Synaptic transmission in APP23 mice has been extensively studied by Rorder and colleagues [51]. They reported that in the prefrontal cortex, synaptic transmission and short-term synaptic plasticity were not altered in APP23 mice compared to age-matched control mice. Notably, the mice were studied up to 24 months of age. In addition, they found that while hippocampal basal synaptic transmission is impaired in APP23 mice, these changes were not associated with alterations in LTP [51]. Overall, these observations are consistent with reports showing no changes in synaptic numbers in 24-month-old APP23 mice [52].

Several groups have tested APP23 mice in a variety of behavioural tests. Cognitive deficits in the MWM were first detected in young mice (3 and 6 months of age, [53] and persisted in 16- and 24-month-old mice [54,55,56]. Spatial learning and memory deficits were confirmed using the water-free Barnes maze [57]. Additionally, working memory deficits are also present in 10-month-old mice [58,59].

A major strength of APP23 mice is the gradual accumulation of Aβ plaques, with widespread plaques appearing by 24 months of age. If the goal is to rapidly screen compounds in vivo for reducing Aβ deposition, other mouse models with higher plaque loads at earlier ages may be more suitable. However, if the objective is to investigate age-related mechanisms contributing to Aβ deposition, the slow progression in these mice makes them an ideal choice. Another distinguishing strength of this model, setting it apart from other APP transgenic models, is the loss of CA1 neurons.

### 2.4. J20 Mice

These mice express human APP695 harbouring the Swedish and Indiana mutations (KM670/671NL and V717F; Figure 1B) under the control of the PDGF-β promoter [60]. They were generated on a C57BL/6 × DBA/2 background and maintained as hemizygous mice. Consistent with the PDGF-β promoter, the mutant APP gene is expressed in neurons throughout the brain [60]. Human Aβ42 levels are readily detectable in the hippocampi of 2- to 4-month-old mice, increasing with age [60]. This increase precedes the formation of extracellular Aβ plaques, which become easily detectable by immunohistochemistry around 6 months of age in the hippocampus and neocortex [60,61]. As the mice age, Aβ deposits become widespread throughout the brain [61,62]. Hippocampal astrogliosis is first evident at 3 months and becomes more pronounced by 6 months [63]. However, this increase plateaus, with no significant difference in astrogliosis observed between 6- and 9-month-old mice [63].

Another key phenotype of J20 mice is the early loss of synapses, which occurs before plaque formation [60,63]. Super-resolution and electron microscopy revealed a ~50% reduction in PSD95-positive puncta in the CA1 stratum radiatum of 3-month-old mice [60,63]. This synaptic loss is followed by deficits in synaptic function. In brain slice recordings from 6-month-old J20 mice, field excitatory postsynaptic potentials were approximately 65% smaller compared to age-matched non-transgenic mice [62]. Consistently, J20 mice also develop LTP deficits [62].

In addition, J20 mice exhibit age-dependent loss of CA1 neurons, as detected by unbiased stereology [63]. Specifically, Wright and colleagues reported a ~10% reduction in CA1 neuronal numbers at 3 months, which increased to ~20% at 6 months and ~30% at 9 months [63]. However, other studies have shown no significant difference in the number of NeuN-positive neurons between J20 and wild-type control mice throughout their lifespan [64].

Given the synaptic deficits and neuronal loss, it is not surprising that J20 mice develop age-dependent cognitive impairments, as reported in numerous publications. For instance, starting at 4 months, J20 mice display spatial learning and memory deficits in the radial arm water maze [63], along with deficits in the Y-maze [63]. These cognitive deficits worsen with age and are evident in other behavioural tasks, including the MWM [62,63,65]. In contrast, both 16- and 20-month-old J20 mice performed comparably to control non-transgenic mice in contextual fear conditioning [63].

A major strength of J20 mice is the extensive electrophysiological characterization available in the literature. As mentioned earlier, these mice develop synaptic dysfunction early in life, prior to the onset of plaques. This makes them highly suitable for studying synaptic alterations associated with soluble Aβ. However, a significant drawback of J20 mice is the high incidence of seizures that occur throughout their lifespan [66,67,68] and the lack of neurofibrillary tangles.

### 2.5. TgCRND8 Mice

These mice express human APP695 harbouring the Swedish and Indiana mutations (KM670/671NL and V717F; Figure 1B) under the control of the PrP promoter [69]. They were generated on various genetic backgrounds, which altered their survival rate: only ~50% of the mice on the (C57BL/6) × (C3H/C57) background were alive at 5 months of age [69]. While early lethality remains an issue, backcrossing the original line to C57BL/6 decreases the mortality rate [70].

Sandwich ELISA data revealed detectable levels of Aβ40 and Aβ42 starting at 4 weeks of age [69]. Between 2 and 6 months of age, there is an exponential increase in Aβ42 levels, with Aβ42 levels being ~500 times higher than those detected at 2 months of age [69,71,72]. Rare Aβ plaques can be detected around 2 months of age [69], and by the time the mice reach 6–8 months of age, plaques become widespread in the neocortex, hippocampus, and olfactory bulb [69,70,71,72,73,74]. Immunohistochemical and biochemical analyses revealed an increase in tau phosphorylation in 12-month-old mice, as detected by the PHF-1, AT100, AT8, and CP13 antibodies [75]. Activated microglia are easily detected around Aβ plaques starting at 3–4 months of age [76]. 

While there have not been reports of frank neurodegeneration in TgCRND8, at 7 months of age, the number of choline acetyltransferase-positive neurons in the nucleus basalis of Meynert was 30% lower than in age-matched wild-type mice [77].

At 3 months of age, TgCRND8 mice show deficits in spatial learning and reference memory, as detected by the MWM and Barnes maze, as well as deficits in cued and contextual memory and object recognition memory [69,78,79,80,81]. These and other cognitive deficits persist as the mice age [69,70,71,78,79,82,83,84].

Due to their accelerated Aβ phenotype, these mice are well-suited for timely assessments of anti-Aβ therapeutic efficacy. However, like many APP transgenic models, they lack significant neurodegeneration and neurofibrillary tangles, which remains a limitation.

### 2.6. 3×Tg-AD Mice

These mice were developed by overexpressing the human APP695 gene harbouring the Swedish mutation and the human tau gene harbouring the P301L mutation, which is associated with frontotemporal dementia with parkinsonism linked to chromosome 17. Both transgenes were under the control of the Thy1.2 promoter and were injected into embryos isolated from PS1-KI mice [85] (Figure 1A–C). The 3×Tg-AD mice were generated on a mixed C57BL/6-129svj background, and hemizygous mice were backcrossed to generate homozygous mice for all three genes. APP and tau levels were 6–8 times higher than endogenous levels, leading to an age-dependent accumulation of Aβ and tau, respectively. Specifically, soluble Aβ42 levels, as detected by sandwich ELISA, rise as early as 4 months of age and gradually increase with age [85,86,87,88,89]. This is followed by the accumulation of Aβ plaques, which can be first detected by IHC in the CA1/subiculum at ~9 months of age. By the time the mice reach 18 months of age, plaques are widespread throughout the neocortex and hippocampal formation [85,86,88,89]. Tau hyperphosphorylation is readily detectable with multiple phospho-tau antibodies in the hippocampus of 3×Tg-AD mice starting at 6 months of age. NFTs begin to appear around 12 months of age and are widespread in the hippocampus by 18 months [85,86,88,89,90,91]. Neuroinflammation coincides with the appearance of extracellular Aβ deposits, around 7 months of age and increases as the mice age [85,92].

While no apparent differences between male and female transgenic mice were observed in the original line described in 2003, over the years, pathology has become more predictable and severe in female mice. In contrast, male 3×Tg-AD mice exhibit a more variable phenotype [93,94]. While it appears that the Thy1.2 promoter expression is higher in female than in male 3×Tg-AD mice [95], this alone cannot account for the phenotypic sex differences that have gradually become evident between them. Notably, early reports found no difference in the steady-state levels of the two transgenes between female and male 3×Tg-AD mice [96]. Furthermore, the stronger promoter activity in female mice does not solely explain the variability in pathology observed among male littermates. Although further studies are needed to fully understand the reasons behind these sex differences, it is plausible that variation in allele segregation occurred during model development across various laboratories. Additionally, an elegant study by Goodwin and colleagues demonstrated that, over generations, transgene copy numbers may vary due to genetic instability at the insertion site [97].

While overt neuronal loss has been a missing hallmark in most animal models of AD, several groups have reported intriguing findings for 3×Tg-AD mice. For example, as early as 2 months of age, 3×Tg-AD mice show enlarged ventricles and alterations in interhemispheric hippocampal connectivity, as detected by diffusion tensor imaging magnetic resonance imaging [98,99]. More interestingly, at 4–5 months of age, these mice exhibit reduced neuronal density and overall grey matter volume in multiple brain regions, including the cortex and hippocampus [89,100]. Consistent with these observations, brain weight is reduced in 5-month-old 3×Tg-AD mice [100].

Several reports have been published on the synaptic dysfunction observed in 3×Tg-AD mice. LTP deficits were reported in the Schaffer collaterals, measured in acute brain slices starting at 3 months of age [101], and persisted through 9 months of age [85,101,102]. These findings are consistent with a decrease in spontaneous inhibitory postsynaptic currents, detected by whole-cell voltage clamp recording [103].

Detailed cognitive assessments of these mice have been performed by several groups. While there are some differences in the literature, there is general agreement on the following time course. The onset of cognitive deficits in 3×Tg-AD mice occurs around 3–4 months of age, affecting multiple cognitive domains [87,89,104,105]. As the mice age, cognition continues to decline. By 6 months of age, these mice show clear impairments in spatial memory and olfactory dysfunction [86,106,107]. At 12 months of age, deficits in the marble-burying test have also been detected [108]. By 18 months of age, 3×Tg-AD mice exhibit profound cognitive deficits across multiple cognitive domains [86,109].

A key strength of 3×Tg-AD mice, setting them apart from other models, is their age-dependent development of neurofibrillary tangles, making them highly suitable for studies examining the mechanisms that regulate interactions between Aβ and tau accumulation. However, it is important to note that tau pathology in these mice is driven by a mutant tau gene. Another limitation is the lack of robust neurodegeneration. Practically, since their initial development, 3×Tg-AD mice have shown a pronounced sex bias: female mice consistently display a stable and predictable phenotype, whereas the phenotype in male mice has become highly variable, even among littermates.

### 2.7. APP/PS1 Mice

The APP/PS1 mice were generated by co-injecting the human APP gene, carrying the Swedish mutation, and the human PS1 gene, carrying the delta E9 mutation (Figure 1A,B), into a single oocyte. Both transgenes were under the control of the PrP promoter [110,111].

These mice develop extracellular Aβ plaques starting at 4–6 months of age [110,112,113], and by 9 months, plaques are readily detected in the hippocampus and cortex [112,113]. A similar progression has been shown in the olfactory bulb, where Aβ deposits are first apparent at 7 months and become progressively more pronounced as the mice age [114,115]. Astrogliosis and activated microglia are first detected at 6 weeks of age [116].

Some degree of neurodegeneration has been reported in APP/PS1 mice. To this end, 12-month-old APP/PS1 mice show hippocampal atrophy, as assessed by MRI [117]. Neuronal loss has been confirmed by others using complementary approaches. For example, stereological assessments indicated a 24% loss of TH-positive neurons in the locus coeruleus of 16–23-month-old female mice [118]. In addition, 24-month-old APP/PS1 mice showed a reduced number of hippocampal neurons, as detected by unbiased stereology, which was associated with an overall reduction in hippocampal volume [115].

Synaptic deficits have also been reported in APP/PS1 mice. To this end, while pre-pathological 2-month-old APP/PS1 mice did not exhibit deficits in synaptic transmission, as the mice aged and AD-like pathology developed, mice showed a marked decrease in excitatory postsynaptic potentials, measured in the Schaffer collaterals using acute brain slices [119,120]. These changes in synaptic transmission were associated with profound deficits in LTP [119,120]. These data are consistent with whole-cell patch clamp recordings conducted in CA3 pyramidal neurons [121]. 

Spatial learning and memory deficits in APP/PS1 mice have been widely documented. Specifically, 8-month-old mice show profound cognitive deficits in spatial learning and memory as assessed by the MWM [122]. As the mice age, cognitive and non-cognitive deficits become more severe and involve several domains. For example, 24-month-old APP/PS1 mice are impaired in the Y-maze, open field, elevated plus maze, social interaction, and social memory [115].

A key strength of these mice is their stable phenotype, particularly in congenic lines, which progresses gradually with age. APP/PS1 mice are, in fact, among the most widely used models in the field. Another advantage is the presence of neuronal loss in older mice, although this loss is limited. This feature makes APP/PS1 mice suitable for studying the interplay between Aβ accumulation, aging, and neuronal loss. However, like many APP transgenic models, a limitation of APP/PS1 mice is the lack of neurofibrillary tangles.

### 2.8. 5×FAD Mice

The 5×FAD mice were developed by Vassar and colleagues in 2006 by overexpressing mutant APP and PS1, harbouring 5 FAD-linked mutations [123]. Specifically, these mice carry three mutations in the APP gene (K670N/M671L, I716V, V717I) and two mutations in the PS1 gene (M146L, L286V; Figure 1A,B). Both transgenes are under the control of the Thy1 promoter. While these mice appear normal at birth, 12- and 18-month-old mice weigh significantly less than age-matched controls [124].

Aβ42 levels, measured by sandwich ELISA, begin to rise at around 2 months of age and show a linear increase as the mice age. Neuropathological examination shows Aβ deposition in the subiculum and cortex as early as 2 months. By 6 months, Aβ deposition is widespread throughout the cortex and hippocampus, with the Aβ load increasing further as the mice age [123]. An increase in microglia number has also been reported in 5×FAD mice, starting at 4 months in the hippocampus and at 8 months in the cortex [124].

Synaptic markers such as PSD95, synaptophysin, and syntaxin, assessed by whole-brain western blot analyses, are significantly decreased in 9-month-old 5×FAD mice compared to age-matched controls [123]. These changes parallel deficits in hippocampal LTP, which were measured in acute slices and detected starting at 4 months [124].

The behavioural phenotype of 5×FAD mice has been extensively characterized. Data from open field analysis show that the walking speed of these mice is significantly reduced at 18 months compared to controls, as reflected by the fact that they cover less distance [124]. Rotarod performance does not correlate with Aβ loads. Specifically, 5×FAD mice are impaired at 4 months but not at 8 or 12 months [124]. A marked decrease in performance in the Y-maze was observed starting at 4 months. Anxiety-like behaviour appears to be task-specific. For instance, while 5×FAD mice are not impaired in the open field, when tested in the elevated plus maze, they exhibit decreased anxiety-like behaviours, spending more time in the open arms and less in the closed arms [124]. Finally, these mice do not show impairment in contextual fear conditioning up to 18 months of age [124].

The 5×FAD mice represent a mouse model in which frank neuronal loss is well accepted in the field. To this end, histological analysis indicated that the number of pyramidal neurons in cortical layer five and subiculum was reduced in 9-month-old 5×FAD compared to age-matched control mice [123]. Consistent with these observations, a striking decrease in neuronal number in cortical layer five has been confirmed by unbiased stereology [123,125,126]. Nevertheless, like with other mouse models of AD, neuronal loss is not present in the hippocampus.

These mice are characterized by an aggressive Aβ phenotype, which does not accurately reflect the chronic and slow accumulation of Aβ deposits observed in humans. Therefore, 5×FAD mice are not suitable for studying the role of aging in the development of an AD-like phenotype. However, the combination of aggressive Aβ pathology and early neuronal loss makes this model highly effective for rapidly identifying interventions aimed at reducing Aβ accumulation in vivo.

### 2.9. APP^NL-G-F/NL-G-F^

A relatively recent mouse model of AD was generated by Saido and colleagues who, using a knock-in approach, replaced the mouse Aβ sequence with the human Aβ sequence harbouring three mutations: KM670/671NL, I716F, and E693G [127] (Figure 1B). Compared to the other mouse models described here, these mice, herein referred to as APP-KI, express physiological levels of mutant APP under the control of the endogenous APP promoter.

**Table 1:** Schematic of the mouse models described here.

Mouse Models	References	Mutant Genes	Mutations
PDAPP	*Games et al., 1995* [23]	APP	V717F
Tg2576	*Ashe et al., 1996* [33]	APP	K670N-M671L
APP23	*Sturchler Pierrat et al., 1997* [44]	APP	K670N-M671L
J20	*Mucke L., 2000* [60]	APP	KM670/671NL, V717F
TgCRND8	*Chishti et al., 2001* [69]	APP	KM670/671NL, V717F
3×Tg-AD	*Oddo et al., 2003* [85]	APP and MAPT, PS1	APP: K670N-M671L MAPT: P301L PS1: M146V
APP/PS1	*Jankowsky et al., 2001* [111]	APP and PS1	APP: K670N-M671L PS1: ∆E9
5×FAD	*Vassar et al., 2006* [123]	APP and PS1	APP: K670N/M671L, I716V, V717I; PS1: M146L, L286V
APP^NL-G-F/NL-G-F^	*Saido et al., 2014* [127]	APP	KM670/671NL, I716F, E693G

APP-KI mice develop age-dependent accumulation of Aβ plaques starting at 6 months of age in the cortex and, to a lesser extent, in the hippocampus [127,128,129,130]. By 12 months of age, Aβ plaques are readily detected throughout the brain [128,129,130]. In general, Aβ deposits are associated with an increase in microgliosis and astrogliosis in brain regions rich in plaques.

LTP deficits were first observed in the prefrontal cortex of APP-KI mice starting at 3-4 months of age, becoming more pronounced as the mice aged [131]. Age-dependent LTP deficits were also present in the CA3-CA1 circuitry [130]. These deficits were associated with a significant increase in both excitatory and inhibitory presynaptic activity, assessed by measuring excitatory and inhibitory post-synaptic potentials, with the latter also showing a modest enhancement in postsynaptic inhibitory function [131]. In addition, spontaneous excitatory synaptic transmission decreased with age [130].

Different research groups have reported behavioural alterations in APP-KI mice. Overall, these mice exhibit reduced exploration activity in an open field, which precedes memory deficits [128,132]. However, in the home cage, the activity ratio during the dark and light cycle was higher in 6-month-old APP-KI mice compared to controls [133]. In addition, 12-month-old APP-KI mice showed higher risk-taking behaviour compared to age-matched controls in the OF test [128], while overall anxiety assessment at 6 months of age showed no differences between APP-KI and control mice [133]. These results, however, are not consistent across research groups, as others have reported decreased anxiety in 6-month-old APP-KI mice [129]. Deficits in contextual fear conditioning were observed starting at 6 months of age and became more pronounced in 12-month-old mice [128,133].

Early deficits in the MWM were evident starting at 12 months of age, although the decline was not extensive [128,129]. In contrast, working memory deficits, assessed by the Y-maze, were evident in 6-month-old female APP-KI mice but not in males [128,129].

A major advantage of these mice, distinguishing them from other animal models, is that there is no overexpression of the human APP gene, as the AD-causing mutations have been knocked into the mouse APP gene. This allows APP to be regulated by its endogenous promoter, resulting in physiological expression levels. In contrast, to develop a robust phenotype, multiple mutations were introduced into the APP gene. Additionally, the absence of neurofibrillary tangles and significant neurodegeneration represents two major drawbacks of these mice.

## 3. Conclusions

Table 2 shows the age of onset of the major neuropathological features of the mice discussed in this review. While AD mouse models have been invaluable in advancing our understanding of the molecular and biochemical mechanisms underlying the disease, they come with both strengths and limitations. One major advantage of mouse models is their amenability to genetic manipulation, enabling precise modelling of genetic risk factors associated with AD. To this end, the Model-AD consortium is actively introducing known risk factors into mouse models of AD, to generate the next generation of more accurate models. Additionally, the relatively short lifespan of mice allows researchers to study disease progression and test therapeutic interventions within a manageable timeframe. Mouse models also offer control over environmental variables, providing a controlled environment that facilitates reproducibility across studies. In contrast, a major drawback is that no single mouse model fully recapitulates the complexity of human AD, particularly its sporadic form. Particularly notable is the inability of mouse models to mimic the profound neurodegeneration that characterizes the human AD brain. Indeed, when neurodegeneration is present in mouse models, it is often mild and not representative of the extensive loss of neurons and brain atrophy characteristic of human AD. While the exact reasons for it are not clear, it is tempting to speculate that mice lack complex genetic and environmental interactions that might be involved in the pathogenesis of AD. For example, laboratory mice are often housed in pathogen-free conditions and fed a controlled diet, and this does not reflect the environment in which people live. The timing of the onset of neurodegeneration is another critical aspect. Many mouse models develop Aβ pathology relatively quickly, often within a few months, while in humans, the progression of AD can span decades. This rapid progression in mice does not allow for the long-term interactions between Aβ, tau, and other environmental and genetic modifiers that are observed in human patients, which may be critical for the insurgence of neurodegeneration. Finally, aging is the major risk factor of AD, even in FAD the disease develops as a function of age. Unfortunately, the aging component is often undervalued during the development of mouse models. Indeed, many models rely on the overexpression of human APP, PS1 or tau, (sometimes harbouring multiple mutations) leading to early and exaggerated pathology that may not accurately reflect the gradual progression observed in humans. Moreover, species-specific differences in brain structure and immune responses raise concerns about the translatability of findings to human AD. Several interventions that successfully reversed AD-like pathology and cognitive deficits in mouse models have failed in clinical trials, highlighting these translational challenges. Thus, while mouse models remain a critical tool, their limitations must be acknowledged, and results should be interpreted cautiously in the context of AD and other human diseases.

## Figures and Tables

**Table 2:** Summary of the age of onset of the AD-like pathology in the indicated AD mouse models. Notably, the age of onset can vary based on genetic background, environmental factors, and specific experimental conditions. Cognitive assessments and the specific methods used can also influence the reported age of onset for cognitive deficits. Abbreviations: mo: months.

Mouse Model	Extracellular Aβ Deposits	Neurofibrillary Tangles	Neuroinflammation	Cognitive Deficits
PDAPP	4 mo	absent	6 mo	3 mo
Tg2576	7–8 mo	absent	10 mo	6 mo
APP23	6 mo	absent	6 mo	3 mo
J20	6 mo	absent	3–6 mo	4 mo
TgCRND8	3–5 mo	absent	3–4 mo	3 mo
3×Tg-AD	9 mo	12 mo	7–9 mo	3–4 mo
APP/PS1	4–6 mo	absent	6 weeks	8 mo
5×FAD	2 mo	absent	4 mo	4 mo
APP^NL-G-F/NL-G-F^	6 mo	absent	6 mo	6 mo

## Data Availability

Not applicable.
